# A Man Who Died of COVID-19 in Karachi

**DOI:** 10.4269/ajtmh.22-0146

**Published:** 2022-06-13

**Authors:** Rabab Batool

**Affiliations:** Aga Khan University, Paediatric Research and Child Health, Karachi, Sind, Pakistan, and Center for Child, Adolescent, and Maternal Health, Faculty of Medicine and Health Technology, Tampere University, Tampere, Finland

Zainab was only 26 when I met her. Although she was well into adulthood, she was not ready to become the most senior adult in her family. Her father, Abbas, was 60 years old, tall, dark, with broad shoulders and big round eyes. He had raised her, her three sisters, and her 17-year-old little brother until he suffered major financial losses. Although Zainab was now running the house, she was not ready to be without Abbas. Three days before he died, Abbas opened his eyes one last time and looked at Zainab. She kissed him on his forehead. It seemed to be a goodbye kiss.

Abbas was first diagnosed with COVID-19 on May 27, 2021. Zainab tried to isolate him at home, but their home was crowded with five adults and one child. He had likely contracted COVID-19 at work or when using public transportation to get to work, where his colleagues could not afford to take days off when they got sick. At home, Zainab checked his blood pressure, sugar levels, and oxygen saturation. In addition to having COVID-19, he had diabetes, hypertension, and Parkinson’s disease. His high-grade fever and weakness from COVID-19 worsened his tremor. Zainab sought out treatments, giving him Panadol, antihypertensive and diabetes medications, azithromycin, dexamethasone, aspirin, and Parkinson’s medications. Zainab had heard that elsewhere in the world people had medications to treat COVID-19 at home. She desperately wanted access to those treatments. However, one night at around 10:30 pm, Abbas’ oxygen saturation dropped, and Zainab brought him to a private hospital, where his oxygen saturation appeared normal. He became more and more somnolent, and had difficulty staying awake. The doctors recommended a computed tomographic scan of his brain, repeat COVID-19 testing, and admission to the isolation ward. Their rates were U.S. dollars (USD) 400 per day, which Zainab and her family could not pay. Considering his condition, the physician advised them to take him to a public hospital.

Hearing Zainab’s story as I conducted an in-person verbal autopsy interview, I could not help but wonder if he would be alive right now if only he had stayed at the private hospital. Can money buy health or prolong life? Although Abbas could not afford the private hospital, other people bought oxygen cylinders and azithromycin to be kept at home in case they got infected. This left hospitals and others struggling and dying because of oxygen shortages. Dexamethasone and toilet paper were also in short supply. The price of remdesivir was too high for someone such as Abbas. In addition, because hospitals charged USD 280 to 850 per day for isolation wards with ventilator facilities, people who could not afford such costs—such as Abbas—did more than suffer. They died.

In the public hospital’s COVID suspect area, Abbas’ portable chest X-ray was fine; his arterial blood gas test results were fine. His oxygen saturation was monitored and seemed to be fine. The physician advised a COVID-19 polymerase chain reaction test again. He also suspected a stroke and sent Abbas to the medicine ward. As Abbas slipped in and out of consciousness, Zainab fed him some tea and biscuits, and later some broth. By the next day, Abbas was too altered to eat. He got bed sores on day 5 after being admitted, and one of his arms started to swell. Then, his oxygen saturation began to drop. As Zainab told me Abbas’ story, I noted he was being treated for stroke and metabolic syndrome—everything except COVID-19. I could not help but wonder, did he get the right treatment? Did he get any antivirals, appropriate antibiotics, good nursing care, or anticoagulants? Moreover, if he had COVID-19, how important was it to keep him in isolation rather than on the medical ward, where he could have infected other patients? Did he suffer from post-COVID pneumonia or was it COVID-19 pneumonia? Despite Abbas’ comorbid diseases, it was clear at this time that, without a ventilator, he was unlikely to survive. His urea and creatine levels were disturbed. His breathing was worsening rapidly. Doctors suspected a pulmonary embolism in addition to COVID-19 because his D-dimer levels were increasing.

Zainab desperately trekked from one ward to another looking for a ventilator. Her legs trembled, her heart beat quickly, and she was sweating profusely in May’s harsh sun, walking from one street to another, one ward to another. There was no ventilator available. Zainab and the doctors discussed moving him to another hospital. As she called her brother to arrange transportation, Abbas died.

During the COVID-19 pandemic, a lot of people suffered from coronavirus disease. Many died and many were cured, but some suffered from the incapacity of the health-care system. Some died because they did not get a ventilator, quality care, or proper management.

As I write out the story of Zainab and Abbas, I ask myself: will we forget those who have lost their lives? Or will we learn our lessons and take appropriate steps to ameliorate some of the COVID-19-related disparities? Should we not try to reach out to the elderly who are isolated in their home, follow up actively on COVID-positive patients through telephone calls, and provide telehealth facilities to them until they recover? People like Abbas, who are poor; people of color, who cannot afford to miss their daily wages, who have to use public transport, and who live in crowded conditions are at greater risk from COVID-19. Like Abbas, many poor workers are more likely to have preexisting health conditions, which puts them at a greater risk of death from the virus. I wonder if we will learn from this terrible illness. Will we fund additional training and capacity development of medical staff and facilities for the poor? Poor or rich, white or black, old or young, each life matters and everyone equally deserves quality of care regardless of their ability to pay.

